# Treatment of pregnancy-related pelvic girdle and/or low back pain after delivery design of a randomized clinical trial within a comprehensive prognostic cohort study [ISRCTN08477490]

**DOI:** 10.1186/1471-2458-4-67

**Published:** 2004-12-24

**Authors:** Caroline HG Bastiaenen, Rob A de Bie, Pieter MJC Wolters, Johan WS Vlaeyen, Janneke M Bastiaanssen, Aldegonda BA Klabbers, Annie Heuts, Piet A van den Brandt, Gerard GM Essed

**Affiliations:** 1 Department of Epidemiology, Maastricht University, P.O.Box 616,6200 MD Maastricht, The Netherlands; 2 Department of Obstetrics and Gynaecology, University Hospital Maastricht, Maastricht, The Netherlands; 3 Department of Medical, Clinical and Experimental Psychology, Maastricht University, The Netherlands; 4 Department of Physiotherapy, Hogeschool Zuyd, Heerlen, The Netherlands; 5 Midwifery practice, Meerssen, The Netherlands

## Abstract

**Background:**

Pregnancy-related pelvic girdle and/or low back pain is a controversial syndrome because insight in etiology and prognosis is lacking. The controversy relates to factors eliciting pain and some prognostic factors such as the interpretation of pain at the symphysis. Recent research about treatment strategies also reflects those various opinions, in fact suggesting there is professional uncertainty about the optimal approach. Currently, physiotherapists often prescribe a pain-contingent treatment regime of relative rest and avoiding several day-to-day activities. Additionally, treatment more often includes an exercise program to guide rectification of the muscle imbalance and alignment of the pelvic girdle. Effectiveness of those interventions is not proven and the majority of the studies are methodologically flawed. Investigators draw particular attention to biomedical factors but there is growing evidence that important prognostic issues such as biopsychosocial factors appear to be even more important as point of action in a treatment program.

**Methods/design:**

This pragmatic randomized controlled trial is designed to evaluate the effectiveness of a tailor-made treatment program with respect to biopsychosocial factors in primary care. The effect of the experimental intervention and usual care are evaluated as they are applied in primary health care. The trial is embedded in a cohort study that is designed as a longitudinal, prospective study, which studies prevalence, etiology, severity and prognosis during pregnancy until one year after delivery. The present paper focuses on choices regarding recruitment procedures, in-/exclusion criteria and the development of a well-timed intervention.

**Discussion:**

This section briefly discusses the actions taken to minimize bias in the design, the proper time-window for the experimental intervention and the contrast between the experimental intervention and usual care.

## Background

Since 1962[1], diagnosis, prognosis and treatment of pregnancy-related pelvic girdle and/or low back pain have inflicted debate and have led to considerable differences of opinions. Many articles appeared mainly in International journals and some etiological mechanisms were hypothesized. However, the subject remains controversial, mainly because insight in etiology and prognosis is lacking. Moreover, diagnostic investigation into the exact definition and classification of pregnancy-related pelvic girdle and/or low back pain shows various opinions between leading experts on this topic. The controversy relates to factors eliciting pain[2] and prognostic factors such as the interpretation of pain at the symphysis [3,4], the question whether pelvic girdle pain is a syndrome separate from low back pain [4,5] and the importance of questions about limitations in activities [6]. Also recent research about treatment strategies reflects those various opinions [7], in fact suggesting there is professional uncertainty about the optimal approach. Investigators draw particular attention to biomedical factors but there is growing evidence that important prognostic issues such as biopsychosocial factors appear to be even more important as basis in a treatment program[8,9]. Although the group of musculoskeletal disorders holds many different biomedical labels, the process of developing chronic disability has shown surprising similarities with regard to biopsychosocial factors [10]. For the moment, pregnancy-related pelvic girdle and/or low back pain is a subjective experience comprising pain and limitations in activities for which classification criteria are insufficient in guiding to a treatment approach (Bastiaenen et al. personal communication). Results of various therapeutic interventions have been published but excepting one recent study[11], their effectiveness remain unproven. Furthermore, the majority of the studies are methodologically flawed [7].

Currently, physicians and physiotherapists usually prescribe a pain contingent treatment regimen of relative (bed) rest and avoiding several day-to-day activities such as using the stairs, bending, twisting, lifting and cycling. Additionally, the usual treatment approach of a physiotherapist more often includes an exercise program to guide rectification of the muscle imbalance and alignment of the pelvic girdle [12]. Therapists rely on knowledge of pain duration and intensity during goal-setting for treatment, for a great deal.

### Why publish a study protocol

There are several reasons for publishing a study protocol before obtaining research data. The main reason is to reflect on the study design independently of the results. Considerations and choices concerning methodology and treatment can be described more detailed. The present paper focuses on choices about recruitment procedures, in-/exclusion criteria and the development of a well-timed experimental intervention. We also present details about the enrollment of women with pregnancy-related pelvic girdle and/or low back pain in the controlled trial.

## Methods/design

### Study design and research question

**Figure 1:**
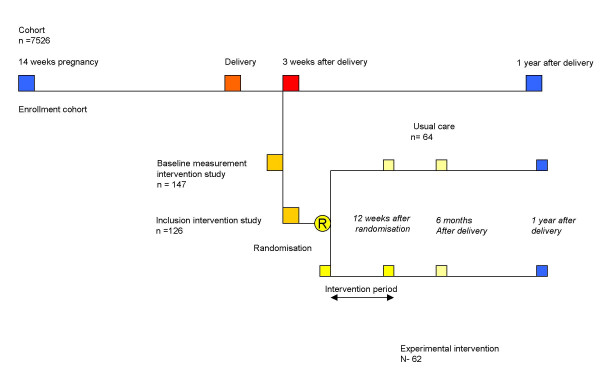
Design of the study

The trial is embedded in a cohort study that is designed as a longitudinal, prospective study, which studies the prevalence, etiology, severity and prognosis of pregnancy-related pelvic girdle and/or low back pain until one year after delivery (Figure 1). The present study is designed as a pragmatic trial aimed to compare the effects of interventions carried out in primary health care.

We performed a randomized controlled trial in primary care to determine whether a tailor-made program with respect to biopsychosocial factors (intervention group) benefits women with pregnancy-related pelvic girdle and/or low back pain more in terms of effectiveness and costs than usual care would on a traditional pain contingent basis (control group).

### Recruitment and informed consent

The medical ethics committee of the Maastricht University Hospital approved the intervention and cohort study. The study is performed in the Southeast of the Netherlands. Midwives and gynecologists recruited the women during early pregnancy (10–14 weeks). Participation of midwives and gynecologists in the recruitment of eligible pregnant women is of major importance for the success of the cohort and intervention study [13]. We have paid a lot of attention to difficulties in recruitment such as busy consultation hours and not feasible recruitment procedures. Therefore, we designed a recruitment protocol that is as simple as possible, not restrictive, and demanding a minimum of time from midwives and gynecologists. Standardized written information about the cohort and intervention study is available for every potentially eligible woman and to be handed out by the midwife or gynecologist. Several steps are taken to encourage participation of the midwives and gynecologists. We distributed newsletters about the developments in the cohort and intervention study every three months and visited the practices and meetings of midwives on a regular basis. Any questions regarding trial questions received prompt feedback. The flexibility of the trial procedure is also guaranteed by assessing potential candidates for the trial at home.

Women are included in the cohort if they are at least 18 years old, pregnant and well versed in Dutch language. Women are given written information explaining the aims and contents of the cohort and intervention study before they decide to participate. Concerning the intervention study they are told that to current knowledge the two investigating treatment options are considered to be equally effective. The moment of inclusion for the intervention study lies at about three weeks after delivery. An individual woman enters the intervention study after signing informed consent for both the cohort and intervention study during early pregnancy and meets the in- and exclusion criteria of the intervention study three weeks after delivery. Women are included when having pain in the pelvic girdle and/or low back with an onset during pregnancy or just after delivery (cohort data), are restricted in their normal daily activities because of pelvic girdle and/or low back pain and if there is a delay in recuperation (not yet in the condition to participate satisfactory in housekeeping and care of children because of the complaints under investigation). The severity of symptoms must be varying with physical activities and time during the day. Women diagnosed with a relevant specific pathology (such as nerve root pathology, rheumatoid disorders, carcinoma, obstetric complications) that affects pain and activities of daily life are excluded. Exclusion also occurs in case of family related or psychosocial problems or when a disablement procedure is not yet finished.

Final important aspects for in-/exclusion are the willingness of a woman to participate in the study or having a clear treatment preference[13]. We only included women who did not indicate such a preference and who were willing to take the 50% risk of receiving a referral to a participating physiotherapist (and treatment option) or the freedom of choosing a therapist by themselves (usual care). Including only women who are naïve (who never received treatment for their complaints during this pregnancy or earlier pregnancies) will result in an unacceptable reduction in the number of eligible patients. However, we excluded all the women who already received treatment after their current delivery.

A basic principle for selection of eligible women in this study is that inclusion criteria must have a meaningful influence in goal setting for treatment. We therefore focused on criteria such as a delay in recuperation and restrictions in normal daily activities caused by pregnancy-related pelvic girdle and/or low back pain. However, other studies in this field formulated inclusion/exclusion criteria based on certain diagnostic classification strategies. Although rationales of these strategies greatly differ, they all attach great importance to the outcomes of particular (albeit different) diagnostic tests. In the absence of a clear definition and reference standard to diagnose pregnancy-related pelvic girdle and/or low back pain, the outcomes of these procedures not only led to different selections of women having complaints, the prognostic and diagnostic importance of these subgroups also remain unclear (Bastiaenen et al. personal communication).

Exclusion of differential diagnoses is a major point of concern. For that reason we included a history taking and a physical examination protocol that focuses on differential diagnoses at first and then on the formulated inclusion criteria. The various specific physical examination tests to diagnose pregnancy-related pelvic girdle pain are left aside. For a better understanding of the complaints and tailoring treatment, application of these tests has no supplemental value (Bastiaenen et al. personal communication).

An experienced research-physiotherapist visited women at home, about three weeks after delivery. This visit is called for on the basis of a short self-administered questionnaire and/or initiated by midwives. A positive answer from a participating woman from the cohort and/or her midwife on the question: "Do you or does this woman need treatment?" took a central position in these questionnaires. In advance of a possible home visit, a short history taking by telephone took place about two weeks after delivery. History taking focuses on exclusion criteria such as: willing to participate in this part of the study, a diagnosis with relevant specific pathology, limitations in daily life caused by pregnancy-related pelvic girdle and/or low back pain and a delay in recuperation. During a home visit, a standardized history is taken and physical examination to exclude specific pathology is performed. Self-administered questionnaires are used to question the women about pain, limitations in activities, restrictions in participation, pain-related fear, pain catastrophizing, positive and negative affectivity, depression, expectancy of treatment result and quality of life. The questionnaires contain clear instructions for completion with no help or support from others. If a woman meets the selection criteria, she is informed about the aim and method of the intervention study and if she is willing to participate, the informed consent procedure is completed. The research-physiotherapist collecting the baseline data is trained in performing the measurements in a standardized way and is unaware of the women's treatment assignments.

### Randomization and blinding

Randomization takes place after collecting the baseline data. In this study we used a block randomization (size of four). An independent research assistant (unaware of the baseline data) carried out the randomization procedure according to a random computer-generated list. When a woman is allocated to the intervention group, the participating physiotherapist in the environment of the woman is contacted and we ensured that treatment could start as soon as possible (within one week). Treatment is covered for all participants in the intervention group on a research-physician's referral. Women, allocated to the usual care group, are free to choose usual treatment by a (not participating) physiotherapist. Information about a possible guidance by a general practitioner and feasible treatments received after randomization is collected by means of questionnaires in the follow-up period.

Women are blinded to a certain extent to the allocated treatment because they are kept naïve of the exact content of both treatment options. Participating physiotherapists are not blinded to the treatment option but not involved in the baseline and effect measurements. Researchers dealing with the baseline and outcome data are unaware of the treatment assignments.

### History and physical examination

During a home visit a standardized history is taken [8] and physical examination is performed. History taking focuses on on-going pain, its location, intensity and modalities, variation of symptoms with physical activities, radiation into the legs, back pain versus leg pain, neurological signs, deformity, obstetric complications, a case history of low back and pelvic girdle pain prior to this pregnancy and other differential diagnoses. The format of the answers is presented as a dichotomous "yes or no". Demographic characteristics and data about education, work, income, use of alcohol, smoking, medication, the onset of pain and functional status during pregnancy have already been gathered as part of the cohort study at 14 and 30 weeks gestation period and two weeks after delivery.

After history taking a short standardized clinical examination program is performed, which includes tests of nerve root radiation (exclusion)[8]. The research-physiotherapist fills out the Pain Behavior Scale, a standardized observation scale for quantifying pain behavior [14,15], after clinical examination.

### Interventions

#### Usual care

Prior to the trial, detailed information is gathered about the contents of traditional treatment options. Part of the information is collected by means of group discussions with experienced physiotherapists and occupational therapists and interviews on an individual basis with affected women out of the cohort. An independent rehabilitation specialist, specialized in pain treatment chaired the meetings with the therapists. Some subjects for discussion were: differences in clinical spectrum seen by the therapists, contents of treatment programs during pregnancy and after delivery, common knowledge by the therapists about etiology, prognosis and prevalence of the syndrome, the optimal time-window for treatment in the course of complaints and the therapist-patient relationship. Items that provided important topics of conversation between the therapists were: the moment of taking up and finishing off treatment, the contents of education and advice given to the patient and the (lack of) compliance. The most striking characteristics of a traditional treatment were the character of the therapist-patient relation and the way of goal setting, focusing on disease management [16]. There was an explicit professional input and an accent on biomedical factors. A pain contingent regimen of avoiding and limiting several day-to-day activities was important. Compliance and adherence based on these goals played an important part. Therapists were often highly concerned about their patient's pain themselves.

However, interviews with affected women made clear that most of the women were irritated about this regimen in an increasing degree after starting the treatment sessions. The regimen was too strict and on a number of points not geared to the wishes and concerns of the women. These aspects caused a lack of compliance and an unremitting hesitation about a good prognosis and in particular about reassuming certain day-to-day activities after delivery. Therapists did not realize the nature of this problem although they did mention problems with compliance. Some women were not able to get a grip on their condition and left management of their pain and activities of daily life to the therapist. A larger part of the women was more or less uncertain about picking up their full range of activities again after delivery. Their beliefs and concerns about origin and prognosis of their complaints clearly bore the stamp of the introduced biomedical label. The relatively favorable prognosis after delivery was largely unknown to the women as well as to the physiotherapists.

#### Experimental therapy

Women, allocated to the intervention group, are referred to a participating physiotherapist in their own neighborhood. These physiotherapists received an educational course about the treatment protocol prior and during the study. All physiotherapists were already experienced and specialized in treating women with pregnancy-related pelvic girdle pain prior to the study. The contents of the experimental therapy are based on the latest literature, results of interviews with affected women (participating in the cohort study) and group discussions with experienced physical and occupational therapists.

A search procedure in literature resulted in various therapeutic interventions. However, effectiveness of those interventions remain unproven. An important common goal of these treatments is restoration of optimal biomechanics, although this is not based on established theoretical principles [7]. The search did not provide enough possibilities to design a treatment protocol. However, as mentioned above, results of interviews and group conversations showed interesting contradictions.

During development of the experimental intervention we focused on the following contradictions: patient-therapist relationship, education, and hesitation or avoiding of activities. Theoretical concepts of self-management [16,17] and fear-avoidance [18] were integrated in the treatment protocol. A treatment program that demands a much more active involvement of a participating woman was designed. Interventions with a self-management approach are considered to be able to build a bridge between patients' needs and caregivers' services to meet those needs. Self-management refers to the individual's ability to manage the symptoms, treatment, physical and psychosocial consequences and life style changes inherent to living with a chronic condition [17]. Self-management approaches are either group-based or individualized. We performed an individualized approach of 7–9 sessions of 30 minutes once a week. Standardized information is presented through a treatment protocol for the therapists and booklets for the patients [16,19]. Topics included back and pelvis anatomy, "red flags" indicating a serious medical condition, factors contributing to fluctuations in pain, appropriate pacing of exercises [12] and activity, handling pain flare-ups, cognitive restructuring, some graded exposure techniques [18,20,21], communication and social persuasion. Therapists had to employ problem-solving techniques that engaged women in identifying day-to-day problems or limitations related to pelvic girdle and/or low back pain, setting personal goals, brainstorming options for achieving these goals and developing personal action plans. In subsequent sessions, women reviewed their action plans and their progress towards goals and engaged in problem-solving skills around difficulties that arose in trying to implement their plans. Information about two opposing behavioral responses of pain-related fear (avoidance and confrontation) is given, and a hierarchy of individual fear-eliciting movements and activities is made. Therapists encouraged women in making action plans for specific activities that were avoided.

Complaint-related problem solving is a key skill. The role of the therapist is to encourage women to identify possible causes of a problem, find a number of potential solutions, select one, then try it and finally evaluate the results and possibly adjust the solution. The second important key skill is action planning or goal setting. Often a plan must have been generally unacceptable (such as "go skiing") for a therapist in the usual care. Nevertheless, the protocol of the experimental intervention embraced the point of view that a woman is her own best judge of what is possible. Another major point of action planning is that a woman could not only receive but also give feedback on her own accomplishments. Endorsement by the therapist is very important for a woman to accept her new role. This way of collaborating with a therapist on short-term action planning enabled women to master new skills and to make changes that are realistic and feasible for them.

Therapists also have a role in assisting women in understanding their symptoms. Knowledge of the course of the complaints during pregnancy and after delivery including pain flare-ups in the year after delivery, factors contributing to fluctuations in pain, evidence-based knowledge about etiology and the concept about pain-related fear are essential. Symptoms are explained as having many but not alarming causes, which offers the possibility to choose different actions by the concerning woman. Finally, therapists have a task in practicing social persuasion. A woman is more likely to change her behavior and have confidence in doing so if she perceives those around her, including the therapist to be supportive.

A relationship in which the physiotherapist and the woman make health care decisions together is the basic assumption of the intervention. Generally, a time contingent policy is followed in which women set the pace by means of action plans. The expertise of the physiotherapists of the condition in general and of the women about their own specific condition and lives are equally important [22].

### Outcome measurements

Outcome measures (Table 1) chosen to explore the success of any intervention need to match the desired aims of that intervention. It is a process in which a standardized attempt is made to observe an often complex clinical picture. Primary domain for improvement of the treatment under investigation is limitations in activities. Other important domains are the severity of the main complaints, the woman's global feeling of recovery, pain and participation.

**Table 1:** Timing of measures

	**Baseline **(about 3 weeks after delivery)	**12 weeks after randomization**	**6 months **(after delivery)	**1 year **(after delivery)
History taking	X			
Physical Examination:	X			
PBS	X			
				
GPE		X	X	X
MC	X	X	X	X
MPQ(VAS)	X	X	X	X
RDQ	X	X	X	X
QBPDS	X	X	X	X
TSK	X	X	X	X
PCS	X			
BDI	X			
NEM	X			
PEM	X			
Expectancy treatment result:	X			
SF-36	X	X	X	X
EuroQol	X	X	X	X
IPA	X	X	X	X
Cost-diary		X	X	X
Satisfaction treatment :			X	X
Recurrence			X	X
Co-interventions			X	
Compliance			X	X
Subsequent pregnancy :			X	X

PBS = Pain Behavior Scale GPE = Global erceived Effect MC = Main Complaint MPQ = McGill Pain Questionnaire RDQ = Roland Disability Questionnaire QBPDS = Quebec Back Pain Questionnaire TSK = Tampa Scale For Kinesiophobia PCS = Pain Catastrophizing Scale BDI = Beck Depression Inventory NEM = Negative Emotionality Scale PEM = Positive Emotionality Scale SF-36 = Short-Form-36 IPA = Impact on Participation and Autonomy

Limitations in activities are measured with the Dutch translation of the Roland Disability Questionnaire (RDQ) [23] and the Quebec Back Pain Disability Scale (QBPDS) [24,25]. We added the phrase "because of my back and/or pelvic pain" in both questionnaires.

Subjective measurements like global feeling of recovery (global perceived effect, GPE) and severity of the main complaints (MC) reflecting a patient-specific approach are also selected. Global Perceived Effect (GPE) is measured by self-assessment on a 7-point scale (1 = completely recovered, 7 = worse than ever). The main complaints (MC) are selected by the woman in a standardized approach by selecting three activities, which are an essential and frequently performed part of her everyday life. However, the performance is difficult or impossible because of low back and/or pelvic girdle complaints at the moment of baseline measurement. Severity of a main complaint is rated on a visual analog scale (VAS). [26,27].

Pain is measured with two VAS-scales of the McGill Pain Questionnaire (MPQ-DLV) [28,29] to record the intensity of pain the last week and day.

The impact on participation and autonomy (IPA) is used to measure person-perceived restriction in participation and autonomy [30,31]. The used subscales are autonomy in self-care, mobility and leisure, social relationships and family role.

Other important prognostic factors that can influence treatment results are fear of movement, pain catastrophizing, depression, negative and positive affect, expectancy of treatment result and pain behavior.

Fear of movement is measured by the Dutch translation of the Tampa Scale for Kinesiophobia(TSK)[32,33]. We used the TSK and the both subscales "fear avoidance" and "harm"[34,35]

Pain catastrophizing is measured by the Pain Catastrophizing Scale (PCS)[36,37].

The Beck Depression Inventory (BDI) [38] measures depressive symptoms [39]. Analyses of the BDI in this study did not include items concerning weight loss, sleeping disturbance and work inhibition [40]

To measure the experience of negative affect we used the 14-item Negative Emotionality Scale (NEM) [41]. To measure positive affect we used the 11-item Positive Emotionality Scale (PEM) [41]. Both are subscales of the Multidimensional Personality Questionnaire.

Health status is evaluated by the Short-Form 36 (SF-36)[42,43] and the EuroQol [44]. We used the subscale "general health".

A cost-diary [45] is used to obtain data on physical activities, health care utilization, and days of sick leave. Women are instructed to record costs on a weekly basis until one year after delivery.

Expectancy of therapy result [46] is measured by means of a 100 mm visual analog scale (VAS). The woman is asked to what extent she believes that a treatment is beneficial to her.

The Pain Behavior Scale (PBS)[14,15] is an observation scale tapping 8 pain behaviors that the physiotherapist completes after physical examination. These are verbal complaints, vocal complaints, facial grimaces, standing posture, mobility, body language, use of visible supportive equipment and stationary movement.

### Follow-up

Women are asked to complete follow-up questionnaires at 12 weeks after randomization, 6 months after delivery and one year after delivery. Women who did not return their follow-up questionnaires were contacted by mail or phone and were asked to continue participation.

### Compliance, other interventions and confounding

The follow-up questionnaires ask all women how many treatment sessions they have followed in the previous period of time. Furthermore, information on contents, satisfaction and the aspects of the (experimental) treatment which benefited them most, is gathered. Co-interventions, medication, aids, additional medical consumption, recurrence of complaints, return to gainful employment and a possible subsequent pregnancy are also registered.

Physiotherapists who treat the participants of the intervention group also answered questions about the number and contents of the treatment sessions after conducting the last meeting.

### Statistical analyses

Statistical analyses are carried out according to the "intention-to-treat" approach. The baseline status of the study groups is compared with respect to the distribution of all independent prognostic variables and the baseline values of the outcome variables. For the outcome measures recorded at baseline and at follow-up, we computed the difference between the baseline and the follow-up score for each woman. Differences between groups and 95%CI are calculated for each outcome measure according to the intention to treat approach. Primary analysis is done by means of analysis of an independent t-test (for continuous outcome variables) and chi-square test (for categorical outcome variables). In order to adjust for possible baseline differences a multiple linear regression analysis for continuous outcome measures is performed with the change scores as dependent variable, treatment option as independent variable and base line scores of the prognostic variables as co-variables. Missing data at the baseline-measurement are substituted by the "mean of series" imputation method. Longitudinal missing data are substituted with the "last value carried forward method". In all comparisons between the two treatment options a two-tailed p-value of 0.05 is considered to indicate statistical significance. Prognostic status at baseline for women with and without missing values for the outcome variables is compared for both groups. Analyses are done by using SPSS statistical software, version 12.0 (SPSS, Inc., Chicago, Illinois). Short term and long term effect analyses are performed separately.

### Economic analyses

A cost-effectiveness analysis compares the costs and health effects of the experimental intervention to assess whether it is beneficial from an economic perspective. The costs of the intervention are calculated separately for the intervention group. For the whole study group all relevant health care costs, production loss and patient and family costs are measured by means of a cost-diary [45] and follow-up questionnaires collected 6 months and one year after delivery. Both direct health care costs (such as physician visits, the number of treatment sessions and medication), direct non-health care costs (such as transport to therapist) and indirect costs associated to the complaints (like sick leave, professional as well as voluntary aid and extra baby sitter) are registered until one year after delivery. Quality of life is measured using the EuroQol. [44]. For the validation of the healthcare costs, patient and family costs, an update of the Dutch manual for costing in economic evaluations is used. The primary outcome measure for the cost-effectiveness analysis is the difference in limitations in activities (RDQ)[23].

### Details about enrollment in the study

**Figure 2:**
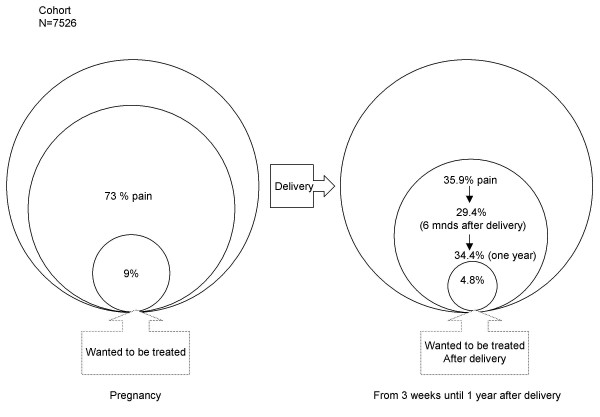
Prevalence of pelvic girdle and/or low back pain during pregnancy and after delivery

During the study, 397 of the 7526 women (5%) signed only for the cohort study (n = 7526) and were therefore beforehand excluded for taking part in the intervention study. Throughout pregnancy, 73% of all women in the cohort reported pain in the lumbar/pelvic region leveling off to 35.9% three weeks after delivery (Figure 2). The "three weeks after delivery" prevalence rate of "wanted to be referred for treatment" was 4.8% at that moment and remained remarkably stable in the year after delivery.

**Figure 3:**
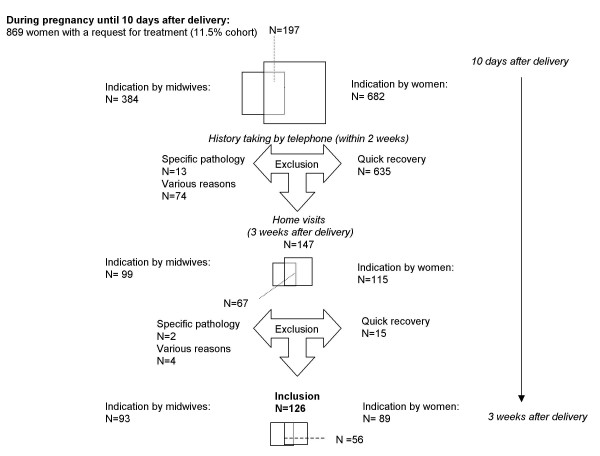
Enrollment in the intervention study

Since November 2000 (Figure 3), 682 women reported that they need treatment during pregnancy (9% of the total cohort). 384 times midwives indicated that a woman need treatment at the time of 10 days after delivery (5% of the total cohort). On 197 occasions, both the woman and her midwife responded positive. The outcomes resulted in 869 possible eligible participants (11.5% of the total cohort). However, these data resulted in only 147 home visits, 99 visits indicated by a midwife (67 times in combination with the woman concerned) and 115 indicated by the woman (Figure 3). On basis of history taking by telephone, 722 women were excluded from participation. Ten women did not give informed consent for the intervention study, 3 women moved outside the area intervention was provided, 13 women were excluded because of specific pathology, 49 women did not want to be randomized (clear treatment preference deviating from the study protocol) and 12 women did not feel like participation on second thought. The majority, 635 women, were excluded because of a quick recovery.

After the home visits, 21 women were excluded. Indicated by a midwife; one because of family reasons, one because of specific pathology and two women because of quick recovery. Indicated by themselves, 17 women were excluded. Two women because of family and social problems, one woman because of specific pathology, one because of a clear treatment preference and 13 women because of quick recovery.

Eventually, 126 women were included in the intervention study. 93 times indicated by midwives, 56 times indicated both by the woman and her midwife and 89 times by themselves. Finally, only 24.2% of the women indicated by a midwife were included and 13% indicated by themselves. Indicated by both the woman and her midwife, 28.4% was included.

## Discussion

This study is designed to evaluate the effectiveness of a tailor-made program with respect to biopsychosocial factors. A pragmatic design provides the opportunity to evaluate the value of the experimental intervention without depriving participating patients of the best current treatment option. Including only women who would take the 50% risk of depriving any treatment at all for their complaints during the first 12 weeks after delivery was not a realistic option. The effects of the experimental intervention and usual care are evaluated as they are applied in primary health care. It is not feasible to blind a woman to the applied treatment option, which increases the risk of information bias. We have tried to minimize this type of bias by assessing treatment preference before randomization and excluding women with a clear treatment preference. Details about the enrollment of the trial underscored this necessity (n = 50 excluded because of a clear treatment preference). The research-physiotherapist dealing with the baseline measurement was therefore unaware of treatment allocation.

Details about the enrollment out of the cohort into the trial also show that the start of the experimental intervention is well timed. Most women have complaints during pregnancy. However, a considerable drop in the number of women having persistent complaints in the first weeks after delivery is observed. Then again, numbers of women having one or more episodes of pain complaints remained stable in the year after delivery. We have seen similar trends of proportions of women with a request for treatment for their complaints during pregnancy (9%), just after delivery (4.8%) and in the year following delivery.

The aim of the experimental intervention is to increase the level of activities. Therefore the primary outcome measure is limitations in activities. The contrast between both interventions is an important issue in this study. Major features that underscore the contrast are the character of the patient-therapist relationship, pain-contingent versus time-contingent treatment, compliance to a regime of avoiding and limiting activities versus action planning and personal goal setting by the women themselves. Among therapists, the approach of the experimental intervention is not widespread at all. The participating therapists are explicitly asked to not give any information about the contents of the experimental treatment to therapists who do not participate in the experimental intervention. It is necessary to interest physiotherapists in the trial for an efficient performing of the experimental treatment option, which can be achieved by a relevant research question and in practice applicable results.

## Competing interests

The author(s) declare that they have no competing interests

## Authors' contributions

CHGB: first author, is involved in the design, data collection, statistical analyses, and the development of the experimental intervention.

RAdB: participated in the design, coordination, statistical analyses, have made substantial contributions to the development of the experimental intervention, is involved in revising the article for important intellectual content.

PMJCW: participated in the design, has made substantial contribution to the development of the experimental intervention and is involved in revising the article for important intellectual content

JWSV: has made substantial contributions to the development of the experimental intervention and is involved in revising the article for important intellectual content

JMB: participated in the design and statistical analyses, is involved in revising the article for important intellectual content

ABAK: participated in the design and collecting of the data

AH: participated in the design, recruitment and collecting of the data

PvdB and GGME; participated in the design, have made substantial contributions to conception, design and experimental intervention and revising the article critically for important intellectual content.

All authors read and approved the final manuscript.

## Pre-publication history

The pre-publication history for this paper can be accessed here:


